# A comprehensive review of metasurface-assisted direction-of-arrival estimation

**DOI:** 10.1515/nanoph-2024-0423

**Published:** 2024-10-21

**Authors:** Min Huang, Ruichen Li, Yijun Zou, Bin Zheng, Chao Qian, Hui Jin, Hongsheng Chen

**Affiliations:** Interdisciplinary Center for Quantum Information, State Key Laboratory of Modern Optical Instrumentation, ZJU-Hangzhou Global Scientific and Technological Innovation Center, Zhejiang University, Hangzhou 310027, China; International Joint Innovation Center, Key Laboratory of Advanced Micro/Nano Electronic Devices & Smart Systems of Zhejiang, The Electromagnetics Academy at Zhejiang University, Zhejiang University, Haining 314400, China; Jinhua Institute of Zhejiang University, Zhejiang University, Jinhua 321099, China; ZJU-UIUC Institute, Interdisciplinary Center for Quantum Information, State Key Laboratory of Extreme Photonics and Instrumentation, Zhejiang University, Hangzhou 310027, China

**Keywords:** direction of arrival estimation, MUSIC and ESPRIT, classical high-resolution subspace methods, compressed sensing (CS) algorithms, machine learning

## Abstract

Direction of arrival (DoA) estimation is a key research focus in array signal processing, and numerous progressive direction-finding algorithms have already been developed. In terms of the development of algorithms, metasurfaces can help innovate traditional estimation algorithms as an excellent alternative to phased arrays. New types of artificial intelligence continue to impact traditional algorithms as well as the detection of the incoming wave direction. Miniaturized and integrated incoming wave estimation devices suitable for various systems have become a significant trend in hardware implementation. In this study, the latest progress and trends in this emerging field are reviewed, and their potential value is assessed. First, a brief overview of a combination of classical DoA algorithms and metasurface is presented. Based on this, the applications of common subspace and sparse representation methods were surveyed, followed by a discussion of their potential prospects. The use of artificial intelligence combined with metasurfaces to innovate DoA detection is discussed. Finally, challenges and opportunities for advancing metasurfaces and artificial intelligence in this frontier field are discussed.

## 1 Introduction

Direction-of-arrival (DoA) estimation is a key technology in wireless communication, astronomical observation, navigation, sonar and radar detection, and medical applications such as emergency services. In particular, it can be used for source localization or tracking by determining the desired locations of the targets in wireless systems, thereby reducing unnecessary noise interference. Traditional algorithms, including the classical Capon algorithm [1], [2], maximum entropy (ME) algorithm [3], [4], high-resolution multiple signal classification (MUSIC) algorithm [5], [6], minimum norm (MN) algorithm [7], estimation of signal parameters via rotational invariance techniques (ESPRIT) [8], [9], and weighted subspace fitting (WSF) [10], have long been employed in DoA estimation. Classical DoA estimation relies on signals collected by multisensor array antennas, which results in complex hardware architectures and high costs [11], [12]. These subspace-based methods and their improvements require eigenvalue decomposition of the covariance matrix of the received signal samples, requiring substantial computational resources and resulting in a low on-site estimation efficiency. Thus, DoA improvements to these established methods are still required. A class of compressed sensing (CS) algorithms based on sparse arrays can effectively reduce the dimensionality of high-dimensional signals, thereby reducing the memory required for storage and transmission. In addition, sparse arrays reduce the power consumption and resource waste. As a novel method for solving complex problems with lower computational requirements, deep learning has garnered significant attention because of its performance in DoA estimation. Therefore, applying existing innovative algorithms to new hardware platforms in a variety of non-ideal environments is a pressing concern.

Over the past few decades, subwavelength structures designed with artificially synthesized metamaterials have been extensively studied by scientific and engineering communities because of their exceptional capabilities in modulating electromagnetic (EM) waves [13], [14], [15], [16], [17], [18], [19], [20]. Metasurfaces, which are the two-dimensional (2D) counterparts of metamaterials, offer several advantages including ultrathin profiles, high integration, and low insertion loss. These attributes enable a more flexible manipulation of EM waves compared to traditional metamaterials [21], [22], [23], [24], [25], [26], [27], [28], [29]. Metasurfaces can be categorized as active or passive types, based on their tunable properties. Passive metasurfaces have fixed phase gradients and can only perform single fixed EM wave manipulations in accordance with the generalized Snell’s law [30], [31], [32]. Methods to achieve tunable performance in metasurfaces include the incorporation of liquid crystals [33], microelectromechanical system (MEMS) switches [34], PIN diodes [35], and varactor diodes [36], [37]. However, active metasurfaces are easily controllable and can be independently managed by field-programmable gate arrays (FPGAs) to modify the phase gradients, thus enabling dynamic and flexible EM wave manipulation. Presently, tunable metasurfaces serve as a powerful and versatile platform for various functions, including programmable holograms [38], [39], EM information theory [40], [41], reflective and emissive arrays [42], [43], vortex beams [44], [45], [46], wireless communication [47], [48], adaptive metasurfaces [49], [50], smart imaging [51], [52], and programmable artificial intelligence machines [53]. Compared with phased arrays or electronically scanned arrays [54], tunable metasurfaces manufactured using printed circuit board (PCB) technology offer comparable performance at a significantly reduced cost and complexity.

The introduction of spatiotemporal and time-varying metasurfaces has introduced new degrees of freedom for controlling EM waves [55], [56], [57], [58], [59], [60], [61], [62]. These innovations have been extensively studied for their potential applications in creating nonreciprocal effects [63], [64], Doppler cloaking [65], [66], frequency shifting [67], and temporal impedance transformers [68]. In fact, by establishing a similar model with metasurfaces that function analogously to antenna arrays, model-driven methods such as the subspace and sparse representation methods can be leveraged to develop low-cost, lightweight DoA estimation devices. Deep learning offers a highly attractive solution for reducing computational complexity and enhancing real-time performance. Deep learning, which utilizes deep neural networks, is a data-driven technology that excels at handling large datasets and solving nonlinear problems. Over the past few years, neural networks have been widely employed to address various complex issues in fields such as mobile communication, big data searches, facial recognition, and artificial intelligence. Once a neural network is trained, minimal computation is required to process new data, making it an efficient tool for real-time applications. Numerous studies have employed deep learning methods to address EM problems, yielding compelling tools for various applications, such as the inverse design of metasurfaces [69], [70], [71], spectrum prediction [72], [73], computational imaging [74], [75], [76], and target detection [77], [78]. The approach for using metasurfaces and deep learning in DoA estimation involves post-sampling of preprocessed array signals to transform the data into a format suitable for neural network input. The data were labeled according to the incident angles, and an appropriate network structure was designed to approximate the relationship between the received signals and the DoA. By utilizing programmable metasurfaces as physical sampling devices and switching through numerous metasurface mode matrices, the signal direction can be estimated from the features of the scattered waves [79], [80], [81]. Another approach based on time-modulated arrays [82] or spatiotemporally modulated metasurfaces [83] determines the signal direction by analyzing the amplitude and phase of the harmonics.

**Figure 1:**
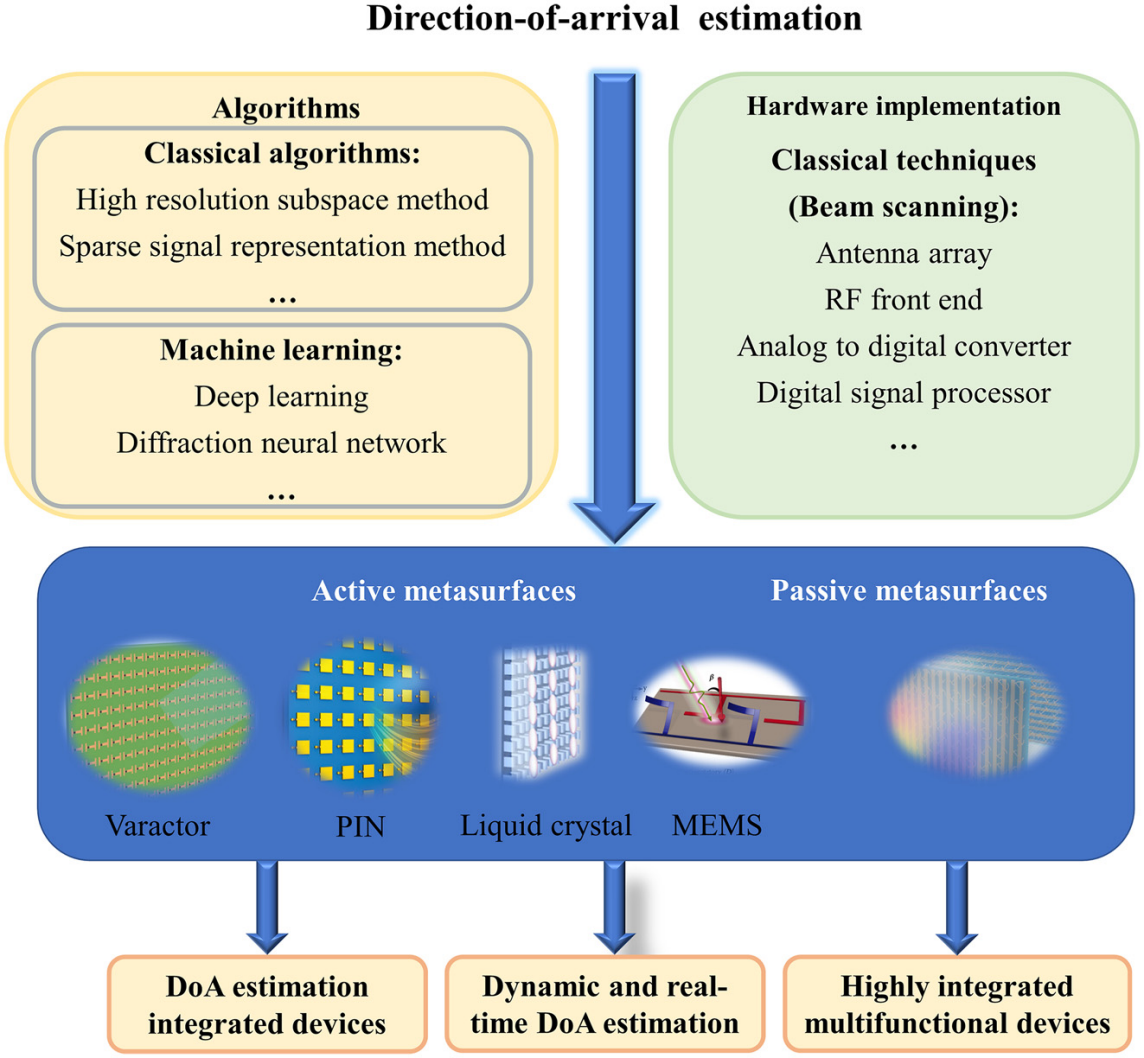
Development of DoA estimation methods based on metasurfaces. With the upsurge of artificial intelligence, DoA estimation methods are mainly divided into two categories: classical analytic methods and machine learning methods. The subspace method and sparse signal representation which are ideally adapted to metasurfaces are considered in the analytical methods. Machine learning algorithms that have been implemented include deep learning and diffraction neural networks. The emergence of metasurfaces brings the possibility of highly integrated multi-function estimation systems and dynamic real-time DoA estimation in the future.

The development of DoA estimation algorithms in conjunction with metasurfaces is reviewed to emphasize implementation methods that integrate classical DoA estimation and machine learning algorithms with metasurface technology (Figure 1). This paper begins by reviewing the fundamental principles and applications of traditional DoA estimation algorithms, followed by a thorough analysis of the unique advantages of metasurfaces for DoA estimation. Furthermore, recent advances in integrating machine learning algorithms with intelligent metasurfaces are discussed, and the significant benefits of these new methods in improving angular resolution, reducing hardware cost, and enhancing system robustness are discussed through theoretical derivation and experimental verification. Finally, a brief outlook is projected towards the prospects of low-cost, highly integrated, real-time dynamic estimation, and multifunctional integrated DoA estimation technologies.

## 2 Classical DoA algorithms for metasurfaces

Metasurfaces are thin arrays composed of electrically small elements with customizable EM properties, enabling exceptional capabilities to manipulate the amplitude, phase, and polarization of EM waves. Consequently, they are widely utilized as low-cost alternatives to phased arrays in applications such as wireless communications, microwave imaging, and millimeter-wave imaging [84], [85]. DoA estimation based on metasurfaces is one application which has garnered significant attention from both academia and industry. Traditional DoA algorithms, including the MUSIC and ESPRIT algorithms, require precise collection of signal amplitudes and phases in phased-array elements [5], [6], [8], [9]. However, tunable metasurfaces can arbitrarily control the amplitude and phase distribution of EM waves, which allows any traditional digital algorithm, such as MUSIC or ESPRIT, to perform DoA estimation. Recent studies on DoA estimation based on metasurfaces have achieved significant progress. In addition to recovering one-dimensional (1D) DoAs from the time delays of incident waves on various elements of the metasurface [86], compressive sensing algorithms have been used to recover DoAs from the random scattering patterns of metasurfaces [79]. The feasibility of these algorithms is validated through numerical simulations and experimental measurements. Additionally, a machine-learning-supported metasurfaces have been demonstrated to recover on-site DoAs from large datasets using complex algorithms [80]. In traditional algorithms, tunable metasurfaces manipulate EM waves through spatial or spatiotemporal modulation to achieve the desired signals, which are then received by devices such as horn antennas or RF links. The modulation coding can be customized based on algorithm requirements, such as using orthogonal coding.

### 2.1 Classical high-resolution subspace methods

**Figure 2:**
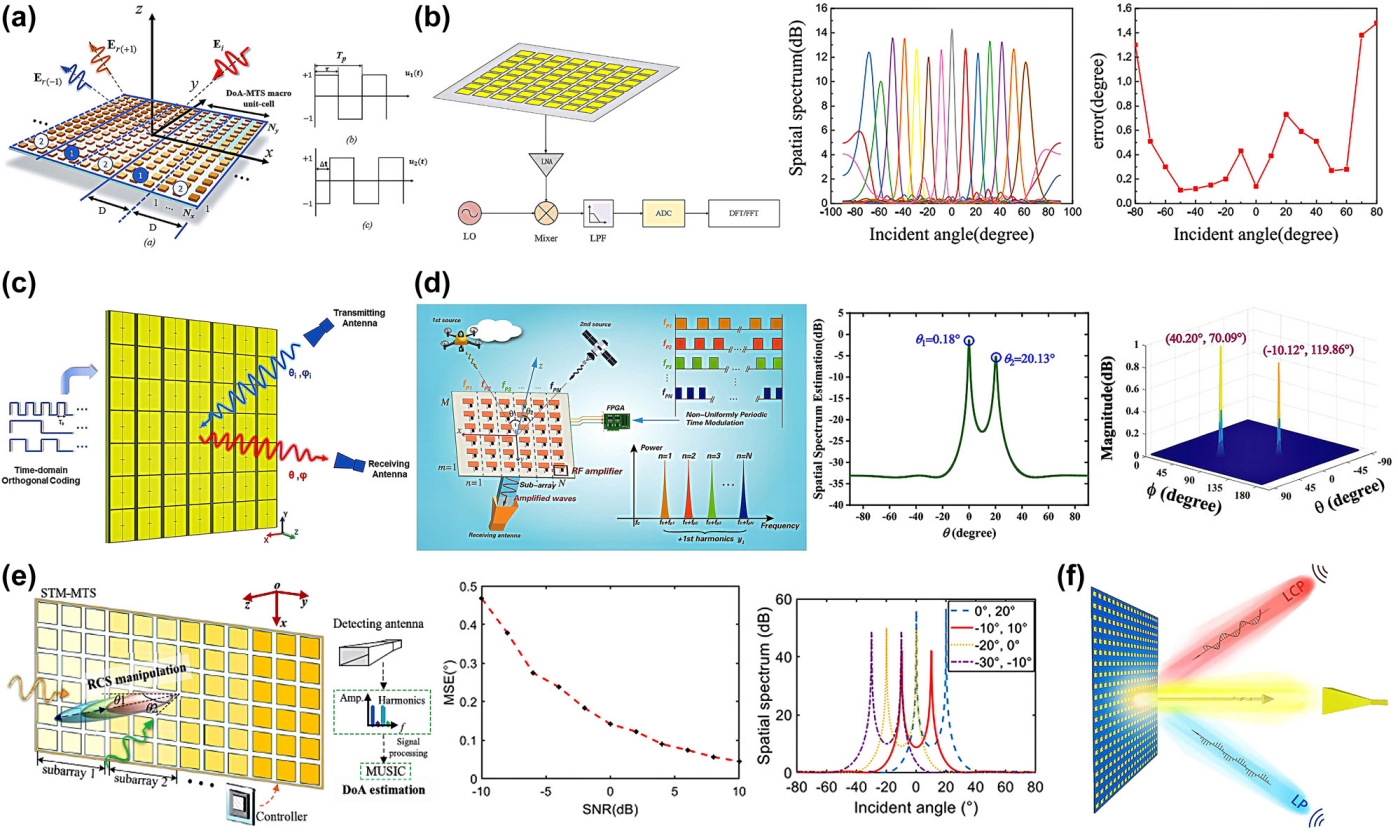
Metasurface DoA estimation method based on high resolution subspace method. (a) Sketch of a space–time modulation scheme on a double-sided band modulating metasurface which can reflect a plane wave formed by the superposition of two first-order harmonics under the irradiation of an incident plane wave. Reprinted with permission from Ref. [83]. Copyright 2022, IEEE. (b) The total received signal is sampled into the digital domain by an analog-to-digital converter, and the coefficients of all harmonics are then estimated using either a discrete Fourier transform or a fast Fourier transform (FFT). Reprinted with permission from Ref. [87]. Copyright 2024, IEEE. (c) Schematic of DoA estimation using a time-domain coded digital metasurface and a single receiver, modulating incident waves by time-domain orthogonal codes, and accurately extracting amplitude and phase distributions from signals detected by receiving antennas. Reprinted with permission from Ref. [88]. Copyright 2022, AIP Publishing. (d) Illustration of the proposed active metasurface for multisource DoA estimation. For 1D applications, the metasurface is divided into *N* subarrays for an equivalent linear array, and each subarray is modulated by their respective frequency. The harmonics generated by each subarray cannot overlap, and the extended spectrum can be applied to recover the multichannel information. Reprinted with permission from Ref. [82]. Copyright 2023, IEEE. (e) The regions used for DoA estimation and manipulation of RCS are independent of each other, ensuring the two functions do not interfere with each other. Reprinted with permission from Ref. [89]. Copyright 2024, IEEE. (f) Schematic of arrival direction and polarization estimation technique based on digital programmable metasurface. Reprinted with permission from Ref. [90]. Copyright 2023, IOP Publishing.

Subspace methods require analysis of the received signals to identify spectral differences and thus determine the DoA. Metasurfaces can flexibly manipulate EM waves, causing continuous changes in scattered EM waves. Thus, tunable metasurfaces can perfectly control spatial variations in EM waves through voltage spatial modulation coding. Scattered waves with different frequencies can be generated by introducing temporal modulation into the coding. Subspace methods are typically adapted for spatiotemporally modulated metasurfaces owing to the additional degrees of freedom provided by spatiotemporal modulation. As shown in Figure 2a, using only the amplitude imbalance in the wide-side reception field at two first-order harmonic frequencies generated by the interaction of the incident plane wave with the modulated metasurface, 1D omnidirectional incident angles can be calculated [83]. Two orthogonally arranged 1D DoA modulation arrays can be extended to 2D DoA estimation, ensuring estimation accuracy while simplifying the computational and hardware complexity.

To further simplify the control of metasurfaces, only time-domain coding can be used to achieve DoA estimations [82], [87], [88]. Without using receiving antennas, the radiation elements and feeding network are integrated. The radio frequency (RF) receiving circuit, built with amplifiers and analog-to-digital converters, performs spectral estimation to achieve 1D DoA detection (Figure 2b and d), further reducing the profile of the direction-finding system [87]. 2D DoA estimation is realized by modulating the incident waves with time-domain orthogonal codes inscribed on the metasurface elements and precisely extracting their amplitude and phase distribution from the signals detected by the receiving antenna (Figure 2b and c). This results in an error of no more than 3° [88]. Reference [82] proposes a DoA estimation method based on amplifier-integrated active metasurfaces that can achieve 2D DoA estimation with only a portion of the metasurface elements, whereas the remaining elements can be redesigned and coded to control the EM waves (Figure 2d and e). This approach allows traditional algorithms such as MUSIC and ESPRIT to be combined perfectly with tunable metasurfaces.

Through simultaneous encoding in the spatial domain, spatiotemporal modulation metasurfaces combined with the MUSIC algorithm can achieve multifunctional synchronization [89], [90], [91]. A DoA estimation system can be developed using spatiotemporal modulation metasurfaces and a detection antenna, where the MUSIC algorithm estimates the spatial spectrum to determine the DoA of an incident wave [89]. This system achieved DoA estimation in the reflection region while controlling the radar cross section (RCS) in the transmission region (Figure 2e and f). In addition, orthogonal metasurfaces were used to estimate the orthogonal components of the incident wave to reduce the computational complexity of the MUSIC algorithm. As shown in Figure 2f, the estimated intensity and phase of these orthogonal components were used to simultaneously estimate the polarization state and direction of the incident wave [90]. With the flexible EM manipulation capabilities of metasurfaces, classical high-resolution subspace methods can be suitably adapted to different application scenarios, reducing the computational complexity and greatly simplifying the hardware requirements [92].

### 2.2 Sparse signal representation methods

**Figure 3:**
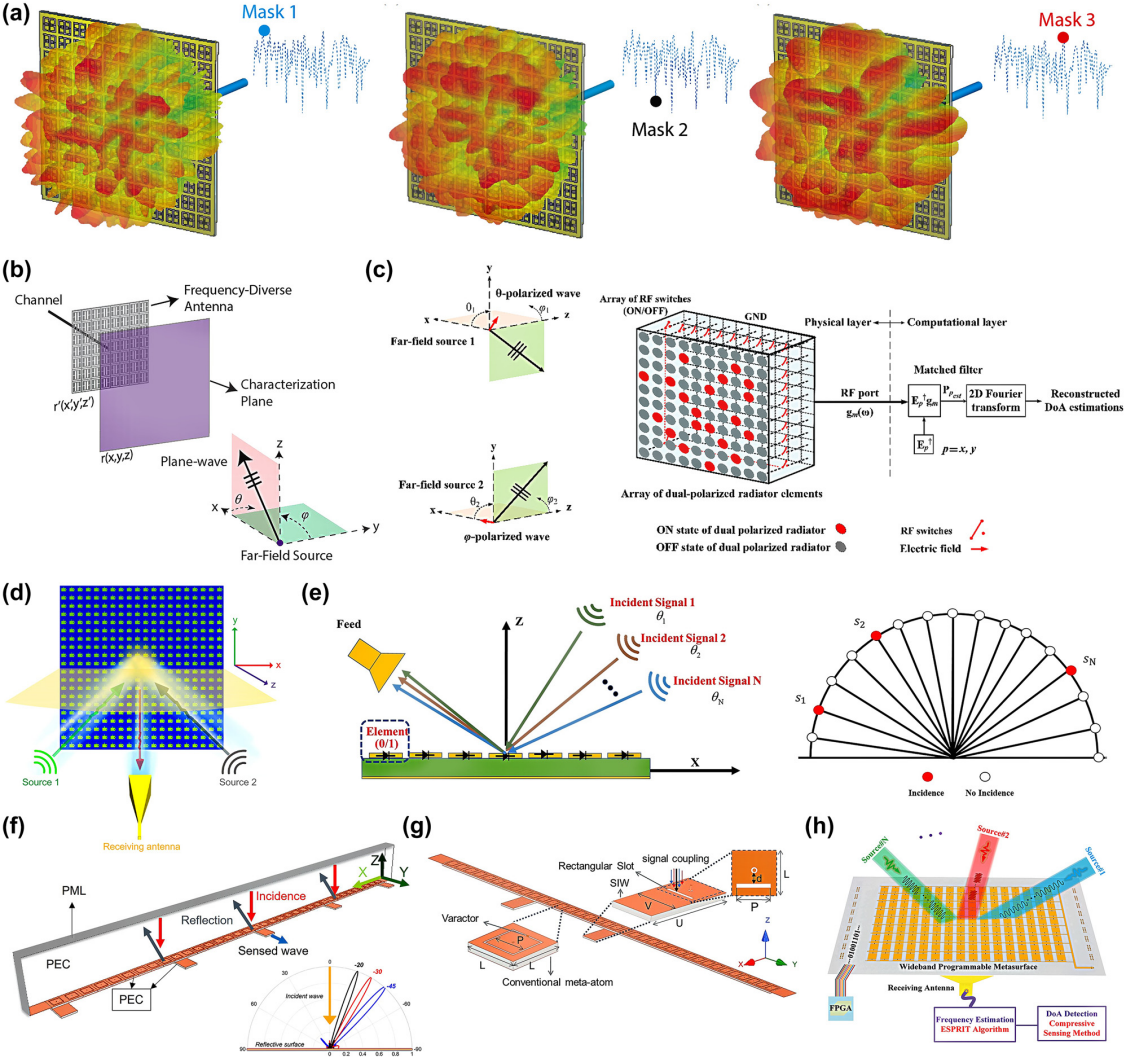
Metasurface DoA estimation method based on sparse signal representation method. (a) Spatiotemporally varying radiation patterns for a wave-chaotic metasurface antenna. The radiation patterns are demonstrated for the first three mask configurations: mask 1, mask 2 and mask 3. Reprinted with permission from Ref. [81]. Copyright 2021, MDPI. (b) DoA estimation using the frequency-diversity technique. The far-field source generates a plane-wave illumination incident on the aperture at (*θ*, *ϕ*). Reprinted with permission from Ref. [93]. Copyright 2019, Nature Publishing Group. (c) The programmable aperture for the polarimetric DoA estimation concepts is developed to react to the change of the polarization of the incoming wave using an array of dual-polarized radiating elements. In the physical layer, the radiation of the elements is controlled by an array of RF switches. Reprinted with permission from Ref. [94]. Copyright 2021, Nature Publishing Group. (d) Schematic of the high precision DoA estimation method based on electronically programmable metasurface. Random dual beams instead of completely random radiation patterns are used in this mechanism to produce the sensing matrix and sample the incident wave. Reprinted with permission from Ref. [98]. Copyright 2021, Wiley-VCH. (e) Space-fed programmable metasurface sensor for DoA estimation and the sparse representation of incident signals. Reprinted with permission from Ref. [79]. Copyright 2021, IEEE. (f) The proposed reconfigurable intelligent surface configuration. Normalized far-field patterns for beams redirected from normal incident on the reconfigurable intelligent surface towards three different desired directions. Reprinted with permission from Ref. [95]. Copyright 2022, IEEE. (g) Proposed architecture for hybrid reconfigurable intelligent surfaces for intensity-only measurement. Reprinted with permission from Ref. [96]. Copyright 2023, IEEE. (h) Schematic of the proposed programmable transmission metasurface to realize the joint detection of the frequency and DoA under the wideband. Reprinted with permission from Ref. [97]. Copyright 2023, IEEE.

Compared with uniform arrays, sparse arrays require fewer elements for the same array aperture, which can reduce the cost of hardware systems and suppress the impact of element coupling on the DoA estimation performance. CS theory offers a novel approach for array signal processing by reconstructing sparse signals with fewer samples. In this theory, signal sampling and compression occur simultaneously, significantly reducing resource waste during signal sampling and transmission. By dividing the entire spatial domain into grids, the signal is sparse relative to the entire spatial domain, allowing CS theory to be applied to DoA estimation and opening new possibilities in the field of DoA estimation. The basic idea of the CS DoA estimation method is to compress the spectra of far-field sources incident on a tunable metasurface aperture into a single channel and then use the transfer function of the metasurface to recover the estimated spectrum [81], [93], [94]. In other words, DoA estimation can be achieved using a simple Fourier transform. For example, the spatial information of the plane-wave projection on the radar aperture is retrieved by utilizing frequency diversity. By performing a simple Fourier transform on this retrieved plane-wave projection pattern (Figure 3a and b), a high-fidelity DoA estimation can be obtained [81], [93]. From the compressed measurements of channel *g*, an estimate of the far-field sources on the metasurface aperture *f*_est_, can be obtained as follows:

(1) $$f_{\text{est}}=H^{+g}$$

*H* denotes the transfer function of the wave-chaotic metasurface antenna. Equation (1) describes a single-shot matched-filtering algorithm that can be applied in real time. Following the estimation of far-field sources on the antenna aperture, DoA estimation can be achieved by applying a simple Fourier transform operation applied to *f*_est_. Extending the concept of frequency diversity to polarization isolation, the same method can accurately detect the (*θ*, *φ*) polarization states of either a single source or multiple sources across a wide frequency range [94]. The orthogonal matching pursuit (OMP) algorithm, which is a greedy algorithm, plays a crucial role in CS reconstruction because of its iterative process based on sparse representation (Figure 3c). Flexible control of metasurfaces can improve reconstruction accuracy. A reflective programmable metasurface was designed to randomly generate a series of dual beams, establish a sensing matrix, and sample the incident waves (Figure 3d). The OMP algorithm was then used to estimate the DoA from a sparsely sampled dataset, achieving error accuracy within 1° for both single and multiple sources [95]. To reduce channel costs and hardware complexity, a method using a tunable metasurface as a control device was proposed [79]. This strategy employs a single sensor to receive different radiation fields at the same location and uses CS to obtain a 2D DoA (Figure 3e), which can be applied under broadband and multi-source conditions. Further improvement in the integration of DoA estimation devices involves the direct use of the metasurface for control and data reception. Reference [95] demonstrates the utilization of inherent information multiplexing within the metasurface substrate, using a sensing RF chain to retrieve relevant information (Figure 3f). This approach ensures low cost and complexity while predicting the DoA of single and multiple incident beams and redirecting the signal to the user. Additionally, using two separate metasurface units to sense the EM wave intensity (Figure 3g) and analyzing the coupled EM wave intensity direction eliminates the need for phase detection and reduces the detection difficulty [96]. In practical applications, the frequency information is crucial and must be detected. Therefore, the joint detection of the frequency and DoA has garnered significant attention. For low-cost simultaneous estimates of frequency information and DoA, a programmable metasurface (Figure 3h) using a single sensor for the joint detection of frequency and DoA over a wide band is appropriate [97]. This method achieved the precise detection of multiple sources at different and identical frequencies, opening new avenues for detecting complex EM environments.

## 3 Machine learning for DoA

**Figure 4:**
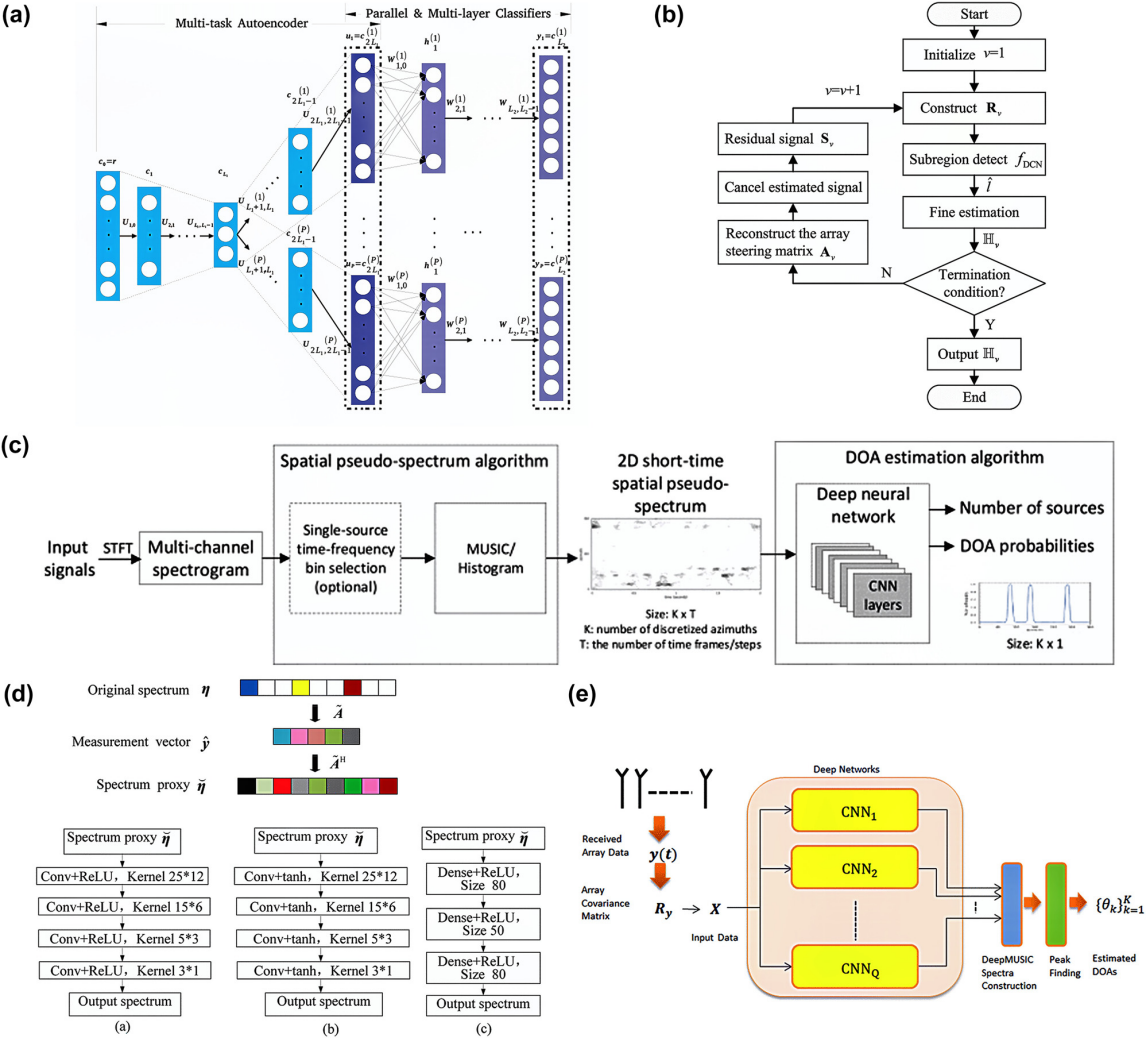
Machine learning for DoA estimation. (a) Structure of proposed deep neural network for DoA estimation. The network consists of two parts: a multitask autoencoder for spatial filtering, and a fully connected multilayer neural network for spatial spectrum estimation. Reprinted with permission from Ref. [99]. Copyright 2018, IEEE. (b) Flow chart of the proposed deep convolution network-assisted 2D multiple DoA estimation method. Reprinted with permission from Ref. [100]. Copyright 2024, IEEE. (c) Block diagram of the proposed DoA estimation algorithm. The spatial pseudo-spectrum algorithm processes the multichannel audio input and produces a 2D short-time spatial spectrum. The 2D convolutional neural network inputs the image-like spectrum and outputs the estimated number of sources and the DoA posterior probabilities. Reprinted with permission from Ref. [101]. Copyright 2020, IEEE. (d) Network structures of the proposed deep convolution network, the counterpart deep convolution network with tanh activation function, and the deep neural network with fully-connected layers. Reprinted with permission from Ref. [102]. Copyright 2019, IEEE. (e) Overall DeepMUSIC framework for DoA estimation. Reprinted with permission from Ref. [103]. Copyright 2020, IEEE.

With the continuous development of deep-learning theories and methods, DoA estimation methods based on deep learning have become a new research direction. Deep-learning-assisted DoA estimation is data-driven and directly builds a mapping relationship between the received data of the array and the angle of reach through the network model, which can effectively solve the unsatisfactory effects caused by array coupling, amplitude and phase errors, and array position errors. Meanwhile, its adaptability and super-resolution in the case of a low signal-to-noise ratio were enhanced, and the computational complexity was reduced. Currently, DoA estimation based on deep learning can be broadly divided into two categories: The first category transforms the DoA estimation problem into a neural network classification problem, classifies spatial angles, and learns the mapping relationship between the network input data and the DoA. For example, in Ref. [99], a multitask autoencoder and a series of parallel multilayer classifiers were employed for DoA estimation, focusing on the robustness of array imperfections, as shown in Figure 4a. Inspired by deep-learning-based DoA estimation and the powerful classification capabilities of deep convolutional networks, a two-stage strategy was proposed, which consists of deep convolutional network classification for region segmentation, followed by successive cancellation-based fine estimation in each subregion [100]. In the first stage, a deep convolutional network with two-dimensional convolutional layers is employed to classify the entire DoA region into multiple discrete subregions of the arrival plane. A K-means clustering algorithm was used to label the training data, thereby enhancing the performance of the deep convolutional network. In the second stage, the OMP method was used to accurately estimate the DoA within each subregion (Figure 4b). For multiple signals, successive cancellation-based fine estimations are performed to refine the DoA estimation. A multitask convolutional neural network using a weighted cross-entropy loss with a 2D spatial pseudospectrum as input features was proposed [101] to robustly estimate the DoA of multiple sound sources in different noise and reverberation environments, even when the number of sound sources is unknown, as shown in Figure 4c. The second category transforms the DoA estimation problem into a neural network regression problem. For example, Wu et al. [102] treated DoA estimation as a sparse linear inverse problem in CS and proposed a deep convolutional network that learns the inverse transform from a large training dataset using sparse priors to effectively obtain the DoA of the signals in real time (Figure 4d). Addressing the limitations of existing studies, where deep learning-based DoA estimation mostly considers only one or two targets, Elbir [103] proposed the DeepMUSIC deep learning framework, which designs multiple deep convolutional neural networks to divide subregions and learn the relationship between the real and imaginary parts of the array received data and their MUSIC spatial spectrum features, thereby providing a DoA estimate for multiple targets (Figure 4e).

**Figure 5:**
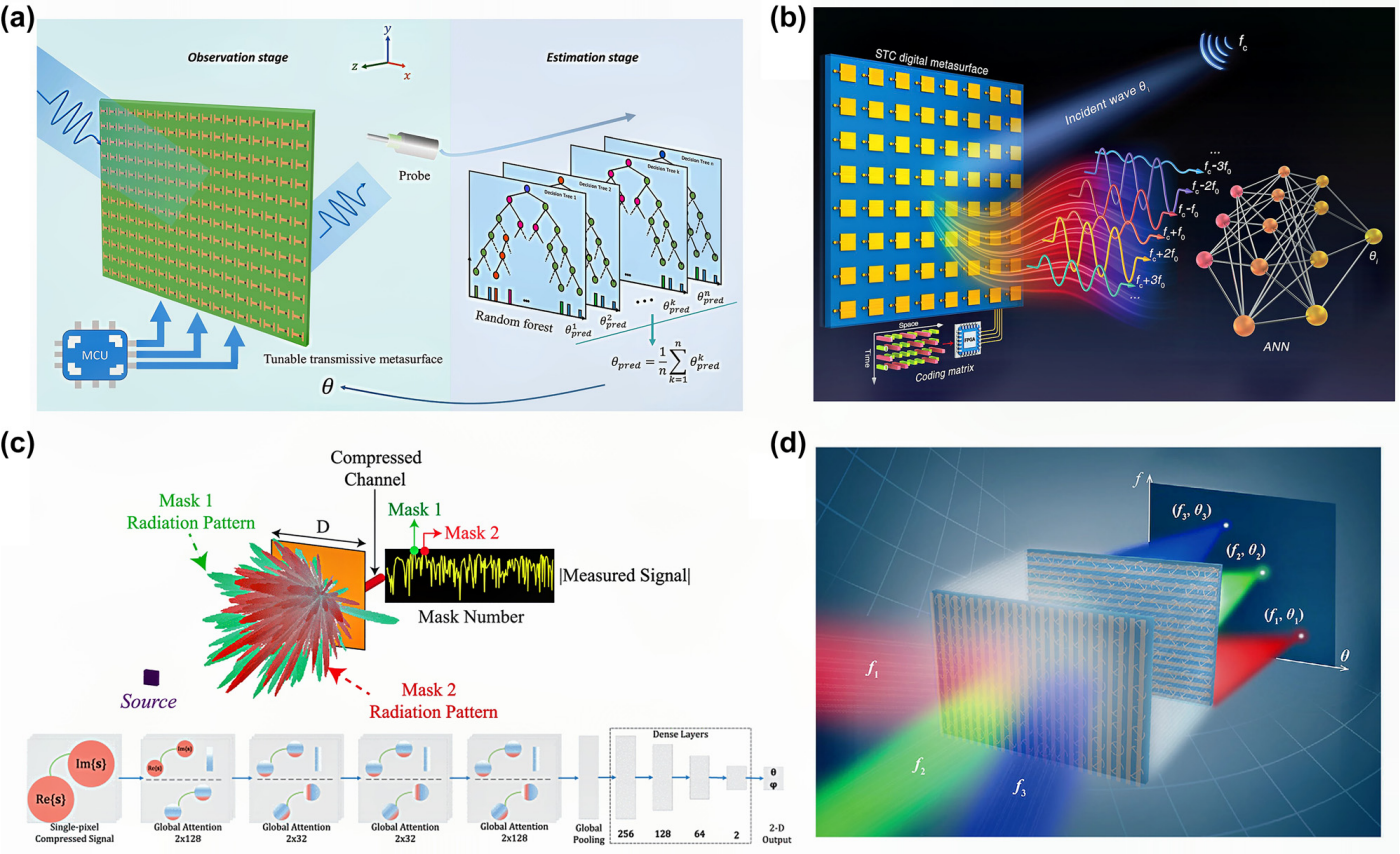
Machine-learning-assisted DoA estimation methods using metasurfaces. (a) Schematic of a machine-learning-enabled metasurface used for DoA estimation. The metasurface-enabled DoA estimation method comprises a tunable transmissive metasurface, one single receiving probe in observation stage, and random forest for prediction in the estimation stage. Reprinted with permission from Ref. [80]. Copyright 2023, De Gruyter. (b) Conceptual illustration of the DoA estimation method based on the space-time-coding digital metasurface and artificial neural network. Reprinted with permission from Ref. [104]. Copyright 2022, Wiley-VCH. (c) Depiction of the coded-aperture-based compressive DoA estimation technique. The proposed graph attention network consists of four graph attention layers and four dense layers respectively for creating feature maps over graph-structured data and regression analysis between features and 2D angle values. Reprinted with permission from Ref. [105]. Copyright 2022, IEEE. (d) Schematic of the multi-source wave-sensing diffraction neural network. Reprinted with permission from Ref. [106]. Copyright 2023, Wiley-VCH.

Neural networks reduce the computational complexity of traditional algorithms, and perform better in noisy and challenging environments. The combination of metasurfaces and neural networks significantly simplifies the hardware. To avoid complex hardware architectures and high costs, Chen et al. [80] used programmable metasurfaces as physical sampling devices. By switching through a large number of metasurface mode matrices, machine learning was used to estimate the signal direction based on the characteristics of the scattered waves, as shown in Figure 5a. During the observation stage, a hardware system consisting of a tunable transmissive metasurface and a single probe was used. The metasurface model was switched multiple times to control the scattering field, and the electric field data were then input into a random forest for estimation decisions, achieving an error of less than 0.5°. Another method based on space-time modulated metasurfaces estimates the signal direction by calculating the amplitude of harmonics, and only relies on a well-designed space-time-coding matrix to convert the incident waves into harmonics which are received by an antenna (Figure 5b). The artificial neural network algorithm considers DoA estimation as a function approximation problem, and establishes a mapping relationship between the normalized amplitudes of multiple harmonics from a single channel and the direction of the signal source [104]. To meet the detection requirements in low signal-to-noise ratio (SNR) environments, a single-pixel compressed DoA estimation technique utilizing a deep-learning framework based on a graph attention network was proposed [105]. The spectra of the far-field sources incident on the aperture are compressed and encoded into the channel of the coded aperture. Deep learning is directly applied to the raw data measured on the compressed channel of a single-pixel receiver, supplanting traditional multichannel grating scanning-based DoA estimation methods (Figure 5c). Graph attention networks enhance DoA estimation in low SNR by focusing on key signal components and suppressing noise, enabling accurate estimation without traditional reconstruction steps. In a multisource detection environment, a highly parallel diffraction neural network composed of a passive metasurface array can simultaneously detect the direction and frequency of multiple incoming waves [106]. The output plane is divided by the frequency and angle, allowing interactions between the incoming waves and the metasurface array to directly display the estimation results (Figure 5d). A multisource diffraction detection system does not require prior knowledge of the number of signal sources, and offers advantages such as light-speed processing, high capacity, and high stability, making it suitable for challenging environments, such as underwater and seismic detection. In summary, the metasurface-assisted intelligent DoA estimation method simplifies hardware by reducing the need for complex components such as T/R modules and RF circuits. Data processing is further enhanced by machine learning algorithms, which decrease computation time and storage requirements. Additionally, diffraction neural networks implemented with metasurface arrays enable real-time operation without the need for data post-processing.

## 4 Integrated computation and detection device for DoA estimation

**Figure 6:**
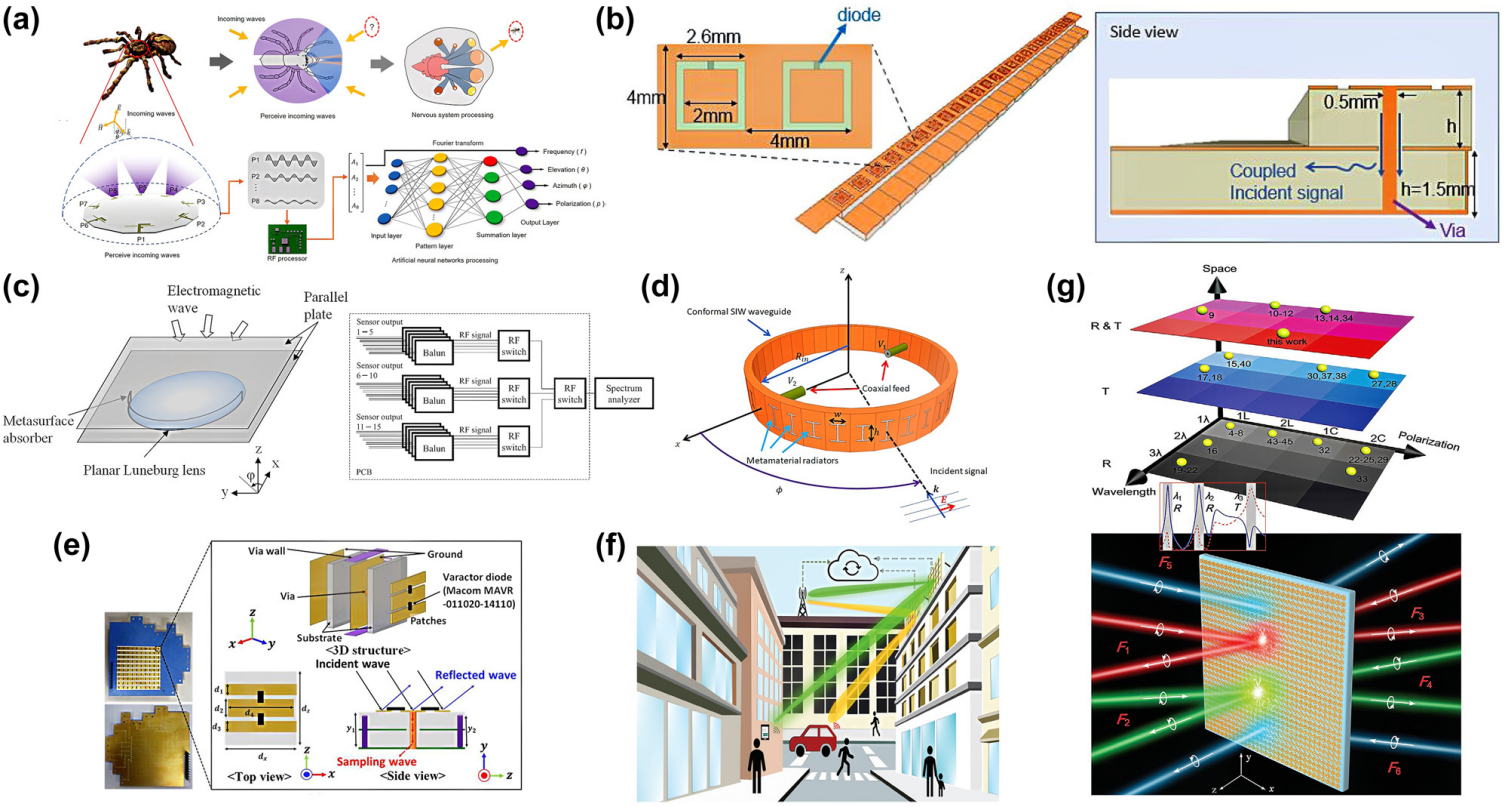
Highly integrated DoA estimation device based on metasurfaces. (a) Schematic of a jumping spider’s visual system and machine-learning-driven incoming wave detector. Reprinted with permission from Ref. [107]. Copyright 2021, Wiley-VCH. (b) Proposed hybrid reconfigurable intelligent surface hardware design with integrated sensing capability. Reprinted with permission from Ref. [108]. Copyright 2021, Nature Publishing Group. (c) DoA estimation concept with planar Luneburg lens and metasurface absorber. Detection circuit on PCB. Reprinted with permission from Ref. [109]. Copyright 2023, IEEE. (d) General configuration of a conformal metasurface antenna with frequency-diverse reception patterns. The resonance frequencies of the metamaterial radiators are randomly selected over a band of operation. The random distribution of resonance frequencies results in patterns that change with frequency and encode information about the DoA of an incident signal into frequency samples, which can then be analyzed to retrieve the incident DoA. Reprinted with permission from Ref. [110]. Copyright 2023, IEEE. (e) Sensing capable reconfigurable intelligent surface unit cell structure. Reprinted with permission from Ref. [111]. Copyright 2023, IEEE. (f) Conceptual illustration of adaptive signal routing control by the connected intelligent metasurface. Reprinted with permission from Ref. [112]. Copyright 2024, Wiley-VCH. (g) Illustration of the evolution and advanced function of the wavelength-direction multitasking Janus metasurface for kaleidoscopic full-space spin-wave control. Reprinted with permission from Ref. [113]. Copyright 2021, Wiley-VCH.

Herein, we introduce DoA detection devices with integrated hardware and algorithms that can be incorporated into the sensing devices. The integrated design can reduce energy loss during signal processing and transmission, thereby improving energy efficiency. Integrated DoA-estimation devices are better suited for space-constrained applications, particularly mobile devices, drones, and small satellites, which require lightweight, compact, and low-power solutions. An intelligent antenna, similar to the structure of a spider’s eye (Figure 6a), can dynamically sense the direction of incoming waves and achieve omnidirectional environmental monitoring [107]. Its unique design and high sensitivity make it applicable in areas such as military surveillance and border security. A reconfigurable intelligent surface with integrated sensing capabilities (Figure 6b) can not only achieve dynamic beamforming through electronic control, but can also sense EM wave information in the environment, thereby optimizing signals and suppressing interference [108]. The integration of sensing capabilities allows for a more efficient utilization of EM wave resources, reducing the overall energy consumption of the system and opening up many future application prospects in intelligent communication networks, such as 5G and 6G networks. A high-precision DoA estimation device utilizing the focal characteristics of a Luneburg lens and the waveguide absorption properties of metasurfaces (Figure 6c) shows significant application potential in high-frequency radar and satellite communications [109]. Frequency-diverse metasurfaces use signals of different frequencies to detect DoAs. As shown in Figure 6d, a flexible frequency-diverse metasurface can detect DoAs from different directions by changing the frequency of the incident signal [110] and providing high-resolution directional information in complex environments. This is crucial for drone navigation and autonomous driving technology. A millimeter-wave reconfigurable intelligent surface device with built-in sensors can automatically track the DoA of signals (Figure 6e), achieving dynamic control of high-frequency signals [111]. This technology has significant value for applications in millimeter-wave communications and future 6G networks, as it can significantly enhance the flexibility and efficiency of communication systems. As shown in Figure 6f, a multitask programmable meta-reflector can support various tasks through programming including signal reflection, DoA estimation, and environmental sensing [112]. Its flexible design makes it widely applicable to smart homes, Internet of Things (IoT), and intelligent transportation systems. A spin-encoded multitask Janus metasurface (Figure 6g) can simultaneously process signals of multiple wavelengths and directions. This multitask processing capability provides significant advantages in complex signal environments, making it suitable for multi-band communication and multi-beam radar systems [113]. Integrated DoA devices outperform existing systems in terms of compactness [114], [115], [116], energy efficiency, performance, real-time capability [117], [118], flexibility, cost-effectiveness, and reliability, making integrated DoA devices widely applicable in modern communication, radar, and navigation fields. To enhance integration with other systems in the future, the DoA estimation component can be designed as an independent subsystem or module, incorporating high-performance data processing chips to minimize latency and bandwidth requirements, thereby enabling real-time responsiveness [119], [120], [121], [122]. In high-frequency bands such as millimeter waves and terahertz, the use of compact metasurfaces will further drive the development of ultra-thin, highly integrated DoA estimation systems that seamlessly combine detection and computation. These advancements will enable more efficient and versatile applications in next-generation technologies.

## 5 Conclusions and perspectives

Here, we provide a survey of the classical DoA estimation algorithm, the latest artificial intelligence algorithm, and a highly integrated metasurface direction-estimation device. The artificial intelligence neural network used for DoA estimation is complete, from theoretical derivation to experimental verification, overcoming the classical algorithm with complex calculations and becoming streamlined and efficient. Hardware architectures have also evolved from high-cost, large-scale antenna arrays to a single metasurface and receiving antenna. The metasurface maintains and improves the performance of DoA estimation in various ways, such as extending the application range from 1D to 2D, from simple directional estimation to estimation of input wave information, and from a single signal to multiple signals, without the need to know the number of signals. The DoA estimation method for microwave frequency band mentioned above can be conveniently extended to higher frequencies. As frequencies shift to higher bands such as terahertz, infrared, and visible light, the shorter wavelengths significantly enhance angular resolution, enabling compact and high-precision DoA estimation for applications like wearable devices and drone navigation. Additionally, with the introduction of new materials like graphene, metasurfaces based on these materials exhibit unique EM properties in the terahertz and infrared bands, enabling fast tuning and dynamic control, which further improves the flexibility and intelligence of DoA estimation systems.

As a novel design and implementation model for metasurfaces, diffractive neural networks (DNNs) have shown unique potential in object classification and recognition [123], [124], [125], logical computation [126], [127], and other fields [128], [129], [130]. By utilizing optical diffraction for signal processing in physical space, DNNs can significantly enhance the real-time performance of DoA estimation. DNNs achieve signal mapping in the direction of arrival/polarization directly through optical diffraction layers, requiring almost no additional computation time. This direct mapping approach enables submillisecond DoA estimation, making it suitable for real-time applications such as autonomous driving, military radar, and fast mobile communications. Furthermore, the optical computations require less energy. When processing signals, DNNs rely mainly on the diffraction and propagation of light, avoiding the complex computations associated with electronic components and significantly reducing energy consumption. This is particularly important for systems with limited power resources such as satellite communications and mobile devices. DoA estimation using DNNs can significantly extend the operational lifetime of the devices and improve the overall efficiency of the system.

**Figure 7:**
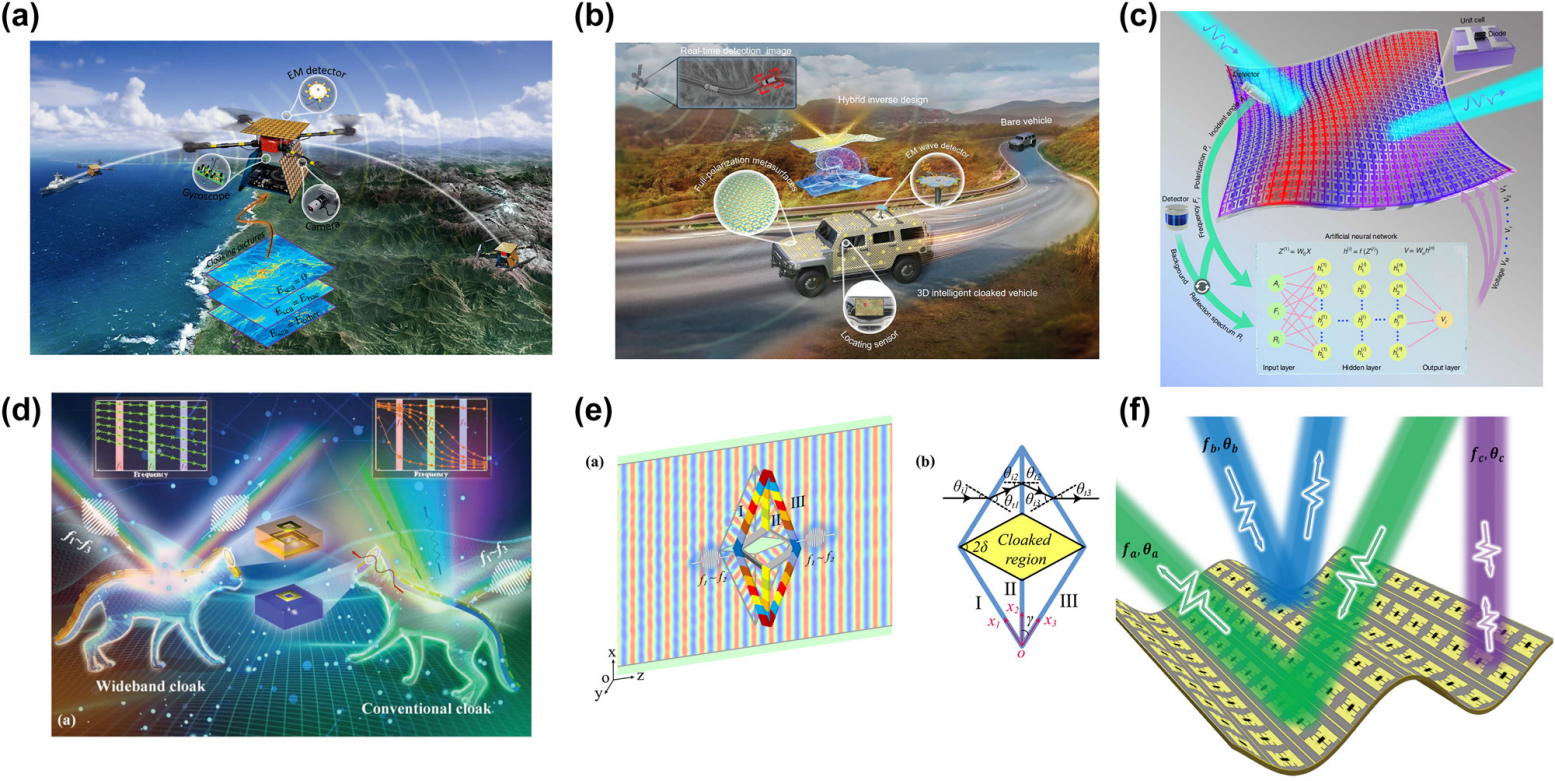
Cloaking system with integrated DoA estimator. (a) The invisible drone integrates sensing, decision-making, and action modules, allowing it to adapt to ever-changing environments and counteract external detection without human intervention. Reprinted with permission from Ref. [131]. Copyright 2024, SPIE; Chinese Laser Press. (b) Smart invisible vehicles equipped with EM wave detectors can monitor the surrounding environment in real time, seamlessly matching with the environment. Reprinted with permission from Ref. [132]. Copyright 2024, Wiley-VCH. (c) A deep-learning-enabled, detector-mounted adaptive metasurface cloak. Reprinted with permission from Ref. [133]. Copyright 2020, Nature Publishing Group. (d) Schematic of wideband metasurface invisibility cloak using material dispersion. Reprinted with permission from Ref. [134]. Copyright 2022, Wiley-VCH. (e) Broadband transmission invisibility cloak. Reprinted with permission from Ref. [22]. Copyright 2024, Wiley-VCH. (f) Schematic of synchronous metasurface invisibility cloak with multiple sources simultaneously incident. Reprinted with permission from Ref. [37]. Copyright 2024, Wiley-VCH.

In the future, there is a foreseeable trend towards integrating these functions into a single system. Frequency, polarization, and direction of arrival are important signal attributes. Multipolarization and multiband information can provide more redundancy, improving the robustness and accuracy of DoA estimation. In environments with multipath propagation and interference, multiband joint processing can effectively distinguish between the direct and reflected waves. Polarization-sensitive antenna arrays can simultaneously measure the polarization characteristics and the DoA of signals, achieving a higher target resolution and stronger interference suppression in complex environments. This multidimensional information fusion approach provides robust technical support for high-precision DoA estimation under challenging conditions. As a fundamental technology, DoA will increasingly integrate with other systems to form multifunctional integrated solutions. For example, in adaptive cloaking systems (Figure 7a–e), DoA estimation can detect the direction and frequency of incoming radar waves in real time by adjusting the EM characteristics of metasurfaces to achieve optimal cloaking effects [131], [132], [133], [134], [135]. In multitarget environments (Figure 7f), DoA technology can help cloaking systems distinguish between different signal sources and adopt different cloaking strategies for various threats [37]. The integration of the DoA technology with EM cloaking systems can significantly enhance their performance and flexibility. With techniques such as high-resolution DoA estimation, multidimensional information fusion, and intelligent cloaking decision-making, future cloaking systems will become more efficient, reliable, and intelligent.
